# Prenatal inflammation causes obesity and abnormal lipid metabolism via impaired energy expenditure in male offspring

**DOI:** 10.1186/s12986-022-00642-y

**Published:** 2022-02-08

**Authors:** Meng Ni, Qianqian Zhang, Jiuru Zhao, Dongting Yao, Tao Wang, Qianwen Shen, Wei Li, Baihe Li, Xiya Ding, Zhiwei Liu

**Affiliations:** 1 Departments of Neonatology, International Peace Maternity and Child Health Hospital, School of Medicine, Shanghai Jiao Tong University, 910# Hengshan Road, Shanghai, 20030 China; 2 International Peace Maternity and Child Health Hospital of China Welfare Institution, Shanghai, China; 3 Shanghai Key Laboratory of Embryo Original Disease, Shanghai, China

**Keywords:** Prenatal inflammation, Obesity, Lipid metabolism, PPAR-γ, Energy expenditure

## Abstract

**Introduction:**

Obesity has becoming a global health issue. Fetus exposed to adversity in the uterine are susceptible to unhealth stimulus in adulthood. Prenatal inflammation is related to poor neonatal outcomes like neurodevelopmental impairments and respiratory complications. Recent studies suggested prenatal lipopolysaccharide (LPS) exposure could result in metabolic disorders. Thus, we hypothesized that offspring exposed to prenatal inflammation could develop into metabolic disorder.

**Methods:**

The pregnant C57BL/6J mice were intraperitoneally injected with 50 μg/kg LPS or saline only once at GD15. The male offspring were weighted weekly until sacrificed. Indirect calorimetry and body composition were both performed at 9 and 18 weeks old. At 20 weeks old, mice were fasted overnight before collecting blood samples and liver for metabolomics analysis and RNA sequencing, respectively. Differentially expressed genes were further verified by RT-qPCR and western blotting.

**Results:**

Prenatal inflammation resulted in obesity with increased fat percentage and decreased energy expenditure in middle-age male offspring. Abnormal lipid accumulation, changes of gene expression profile and upregulation of multi-component mechanistic target of rapamycin complex 1 (mTOR)/Peroxisome proliferator-activated receptor-γ pathway was observed in liver, accompanied with decreased bile acids level, unsaturated fatty acids androgens and prostaglandins in serum. Indirect calorimetry showed increased respiratory exchange rate and deceased spontaneous activity at 9 weeks in LPS group. Impaired energy expenditure was also observed at 18 weeks in LPS group.

**Conclusion:**

Prenatal LPS exposure led to obesity and abnormal lipid metabolism through impaired energy expenditure.

**Supplementary Information:**

The online version contains supplementary material available at 10.1186/s12986-022-00642-y.

## Introduction

Obesity affects a third of the world population [1, 2], and it disturbs nearly all physiological functions of the body. Obesity increases the risk of developing multiple disease conditions, such as diabetes mellitus [3], cardiovascular disease [4], several types of cancers [5] and poor mental health [6]. Despite the risk factors as heredity and western diet [7], a clear understanding of the potential mechanisms originated from early life which might permanently program the metabolism is still lacking. According to the developmental origins of health and disease (DOHaD) theory, fetus exposed to adversity like toxins, malnutrition, infection and other none-infectious inflammation in the uterine are susceptible to stimulus in adulthood [8, 9].

According to research, 63.6% of women reported at least one infection during pregnancy, with 49.6% reported a respiratory infection, 20.5% a fever and 17.1% a urinary tract infection [10]. Therefore, low levels exposure of LPS through Gram-negative bacteria infection [11] is a common condition in pregnant women [12]. Previous studies showed that prenatal LPS exposure could result in prematurity, abortion [13, 14] and intrauterine growth restriction [15]. Furthermore, metabolic disorders such as glucose tolerance impairment, insulin resistance [16] and obesity were observed in offspring exposed to prenatal LPS exposure [17–19]. However, the other metabolic disorders and potential mechanism beneath it remain to be elucidated.

In the study, we aimed to investigate whether prenatal LPS exposure led to metabolic disorders in offspring and the potential mechanism might contribute to it.

## Materials and methods

### Animals and treatments

The female C57BL/6J mice (6 weeks old, 16–18 g) were purchased from Shanghai Laboratory Animal Co. Ltd. (SLAC, Shanghai, China). Animals were maintained in specific pathogen free (SPF) environment with a 12-h cycle (light on, 7:00 a.m.; light off, 7:00 p.m.), a constant temperature (20–25 °C), and food and water were attained voluntarily. Females were mated with males (2: 1) at 5:00 p.m. overnight, and the presence of a vaginal plug the next morning was confirmed as gestational day (GD) 0. Pregnant mice were divided randomly into LPS or saline group. The dose–response effect of LPS (Sigma Chemical Co., St. Louis, MO, USA) was evaluated in pregnant mice (Additional file 1: Table S1) in a pilot experiment to optimize the concentration and time window of exposure. A dose of 50 µg/kg LPS at GD15 for a one-time exposure was confirmed with an acceptable survival rate of pups without significant change compared with the rate of saline group (Additional file 1: Figure S1, Table S2). After delivery, the pups were randomly discarded to keep the litter size as five pups per cage. At weaning, we combined pups from different litters of the same maternal treatment for further study. Only male pups were kept for analyses in following study, since obesity was not observed in female in the pilot study (not shown). Maternal and neonatal baseline characters were described (Additional file 1: Table S2).

**Fig. 1:**
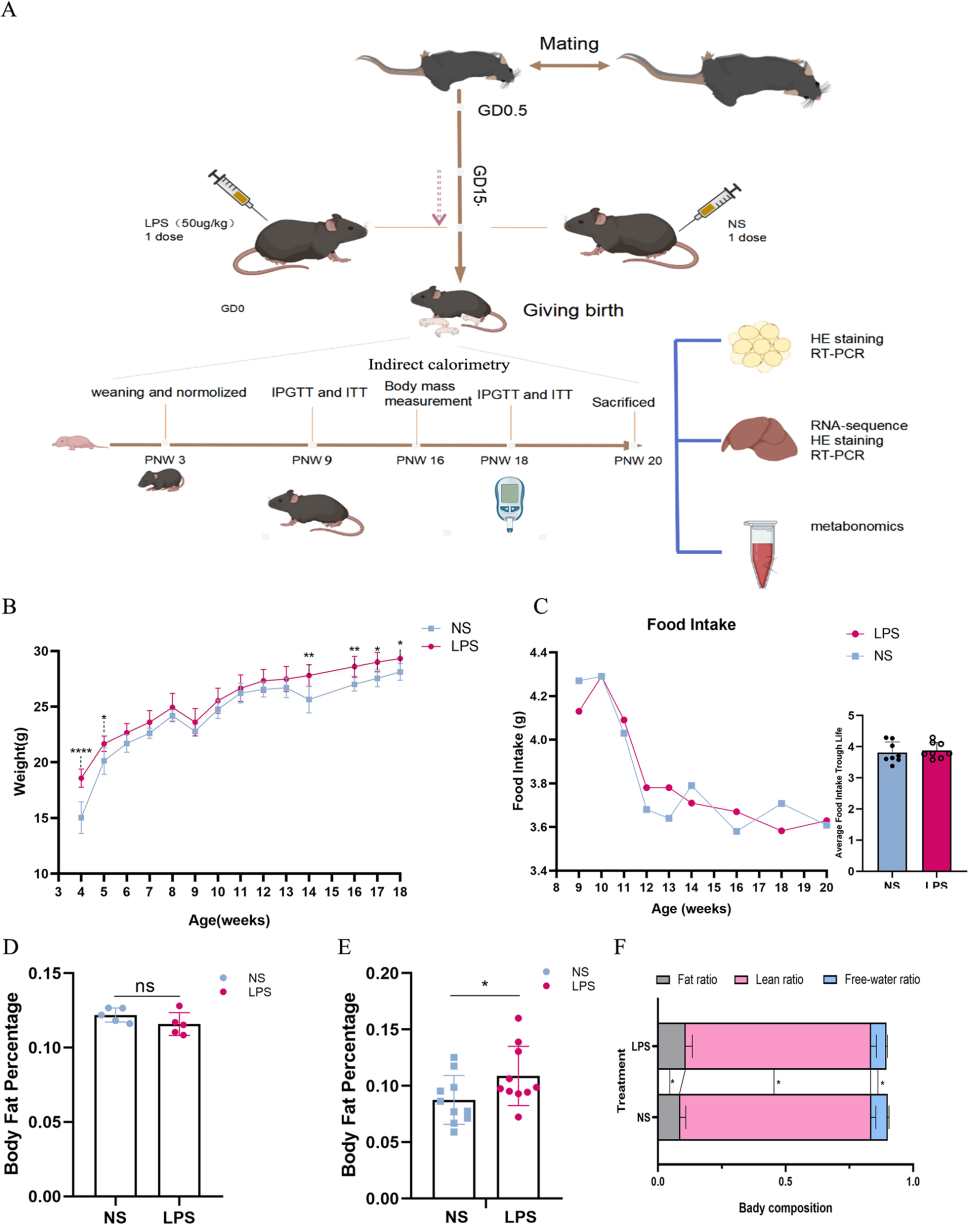
**A** The flow chart of the experiment. **B** The body weight of male offspring through life span (LPS: n = 12; NS: n = 10). **C** Food intake of the male offspring (Left) and average food take trough life (Right). **D** Body fat percentage at 9 weeks (LPS: n = 6; NS: n = 5). **E** Body fat percentage and **F** Body composition at 18 weeks (LPS: n = 12; NS: n = 10)

The offspring were weighted weekly until sacrificed. Indirect calorimetry was performed at 9 and 18 weeks old, and body composition was measured at 9 and 18 weeks old. Intraperitoneal glucose tolerance tests (IPGTTs) and insulin tolerance tests (ITTs) were both performed at 9 and 18 weeks old. Mice, which were fasted overnight beforehand, were anesthetized using isoflurane and euthanized via cervical dislocation at 20 weeks old. Blood samples were collected, and then kept at 22–26 °C for 2 h before centrifugation at 4 °C and 5000 rpm for 10 min for the analysis. Liver, retroperitoneal and gonad fat were quickly weighed and collected in liquid nitrogen for further RNA and protein preparations, and then stored at − 80 °C (Fig. 1A).

### Food intake

The offspring’s daily caloric intake was measured once per week from 6 to 18 weeks of age during the experiments using the following equation:

$$Average\;food\;intake = \frac{{food\;weight \left( {before} \right) - food\; weight \left( {after} \right)}}{days \times number\; of\; pup}$$

### Body composition analysis

The body composition of the offspring was assessed at 9 and 18 weeks of age using magnetic resonance imaging (EchoMRI 900, Houston, TX, USA), according to the manufacturer’s instructions. Fat mass (g), lean mass (g) and free-water mass were measured and calculated as percentage.

### Indirect calorimetry

At 9 and 18 weeks old, the offspring were measured for 24 h by compressive animal lab animal monitoring system (Oxymax-CLAMS, Columbus Instruments, Columbus, OH, USA). Gas mixture was calibrated before the experiments. The animals were placed individually in a single cage chamber and maintained for 12 h in advance to avoid anxiety. Oxygen consumption, carbon dioxide release, energy expenditure, and respiratory exchange ratio (RER) data were collected. The following equations were used for the calculation of corresponding parameters [20]:

$$Energy expenditure \left( {EE;W, \frac{J}{s}} \right) = \frac{{16.3 \times VO_{2} + 4.57 \times VCO_{2} }}{60}$$

$$Lipid oxidation \left( {L^{OX} , W} \right) = \left( {1.69 \times VO_{2} - 1.69 \times VCO_{2} } \right) \times \left( {\frac{9.46 \times 4.186}{{60}}} \right)$$

$$Glucose oxidation \left( {G^{OX} ,W} \right) = \left( {4.57 \times VCO_{2} - 3.23 \times VO_{2} } \right) \times \left( {\frac{3.74 \times 4.186}{{60}}} \right).$$

Total activity was evaluated by adding the spontaneous activity (X-Amb) and rearing activity (Z-Tot).

### IPGTT and ITT

For the IPGTT, the mice were fasted overnight (from 5:00 p.m. to 9:00 a.m.). Each received an intraperitoneal injection of glucose (2 g/kg). The glucose concentration was measured from the tail at certain times (0, 15, 30, 60, 90, and 120 min) using a glucometer (Roche Accu-Chek Inform, Switzerland). For the ITT, the mice were fasted for 6 h (from 8:0 a.m. to 2:00 p.m.). Each received an intraperitoneal injection of insulin (0.75 U/kg), and the blood glucose was measured at certain time points described above. The tests were performed at 9 weeks old and 18 weeks old.

### Histological analysis of liver and adipocytes

The dissected liver and adipose tissues were fixed with 4% paraformaldehyde in phosphate-buffered saline for 16 h at 4 °C, sequentially dehydrated and embedded in paraffin. The tissue samples were then sectioned at a 6 μm thickness for hematoxylin–eosin staining and quantification. For the Oil Red O staining, the sections were incubated in 60% isopropyl alcohol, and then stained with filtered Oil Red O solution (3 mg/mL) for 30 min and rinsed twice with distilled water. Images were obtained (Nikon Eclipse Ti microscope, Tokyo, Japan) and quantified (Image J software, USA). Five areas for calculation of the area under the curve (AUC) were randomly chosen at the interval of every two visual fields under 400X amplification.

### Quantitative real-time polymerase chain reaction (qPCR)

The total RNA was extracted from liver and fat tissues with Trizol reagent (Takara, Shiga, Japan). Then, quantitative real-time reverse-transcription PCR was conducted using SYBR green real-time PCR master mix reagent (Toyobo, Osaka, Japan) on the platform of QuantStudio 3 Real-time PCR System (Life Technologies, Inc., Carlsbad, CA, USA). This method was performed for all samples in triplicates. The primers are listed in Additional file 1: Table S3.

### Western blotting

The total protein was extracted with RIPA (EpiZyme, Shanghai, China), according to the manufacturer’s instruction. Then, the protein lysates were separated by sodium dodecyl sulfate–polyacrylamide gel electrophoresis (SDS-PAGE) and transferred to a polyvinylidene fluoride membrane (Millipore, Darmstadt, Germany). The membrane was incubated overnight with specific antibodies at 1:1000 at 4 °C. The primary antibodies used were β-actin (#3700), phosphorylated (p)-mTOR (Ser2448) (# 5536S), total (t)-mTOR (#2983) and Peroxisome proliferator-activated receptor-γ (PPAR-γ) (#2435 T) (Cell Signaling Technology, MA, US). The chemiluminescent assay kit (EpiZyme, Shanghai, China) was used for color reaction.

### Measurement of lipid in mice liver

The total cholesterol (TC) (A110-1-1) and triglyceride (TG) (A111-1-1) in the mice liver were extracted with absolute ethyl alcohol and measured by commercial kits, according to the manufacturer’s instructions (Jiancheng Bioengineering Institute, Nanjing, China). The TC and TG levels were determined from the optical density of each well at 510 nm wavelength using a microplate reader (Synergy H1, Biotek, VT, USA).

### ELISA analysis

All ELISA kits, including TNF-α (ml002295), IL-6 (ml002293), IL-1β (ml06132) and HbA1c (ml401824) were purchased from Enzyme-linked Biotechnology (Shanghai, China.). Sample preparation and ELISA assays were performed following the instructions of manufacturer. Each extract was analyzed in triplicate wells in a 96-well microtiter plate. Optical density was read at 450 nm wavelength using a microplate reader (Synergy H1, Biotek, VT, USA).

### Metabolomics analysis

#### Sample preparation

All chemicals were analytical or high-performance liquid chromatography-grade with water, methanol, formic acid, acetonitrile from (CNW Technologies GmbH Düsseldorf, Germany) and L-2-chlorophenylalanine from (Hengchuang Bio-technology Co., Ltd., Shanghai, China). The samples stored at − 80 °C were thawed at 22–26 °C and prepared as previous researches described [21].

#### Data processing and statistical analysis

Progenesis QI software (Waters Corporation,Milford, CT, USA) was applied for the raw data of LC–MS. Parameters were set as followed with the precursor tolerance as 5 ppm, the fragment tolerance as 10 ppm and the retention time (RT) tolerance as 0.02 min. Isotopic peaks were determined according to peak RT alignment in internal standard detection. 15% of the base peak intensity was set to identify the minimum response. The parameters included *m*/*z*, peak RT, peak intensities while RT-*m*/*z* pairs were applied for identify the ion. After trimming samples with missing values (ion intensity = 0), less than 50% of the samples were finally included. The metabolites were mapped to the database (http://www.hmdb.ca/; http://www.lipidmaps.org/; and self-built databases) Progenesis QI (Waters Corporation) was used for the data analysis. The data were processed by the R package ‘ropls’. Mean centering (Ctr) and Pareto variance (Par) were applied for initial data processing to visualize the alterations by principal component analysis (PCA) and (orthogonal) partial least-squares-discriminant analysis (O)PLS-DA, respectively. The Hotelling’s T^2^ region was used to define the 95% confidence interval of the variation. Variable importance in the projection (VIP) > 1 are selected for discrimination. In this study, after one-seventh of the samples was excluding from the model in each round where the default seven-round cross-validation was applied to avoid overfitting. Metabolites with VIP values > 1.0 and *P* < 0.05 were remained as differential metabolites. Hierarchical clustering and correlation analysis were performed on top 50 statistically different metabolites. Kyoto Encyclopedia of Genes and Genomes (KEGG) enrichment was performed to identify the pathways. Enrichment with q value < 0.05 was considered significant.

For lipid analysis, differential metabolites were identified with VIP > 1 and *P* < 0.1. KEGG enrichment was performed using MetaboAnalyst 5.0 (https://www.metaboanalyst.ca/).

### RNA sequencing

The total RNA of liver was extracted with RNAiso Plus Total RNA extraction reagent (Cat#9109, TAKARA), as instructions mentioned. RNA integrity was validated referring RIN number through an Agilent Bioanalyzer 2100 (Agilent technologies, Santa Clara, CA, USA). Then, qualified total RNA was purified with RNA Clean XP Kit (Cat A63987, Beckman Coulter, Inc. Kraemer Boulevard Brea, CA, USA) and RNase-Free DNase Set (Cat#79254, QIAGEN, GmBH, Germany).The library was constructed with the TruSeq Stranded mRNA LT Sample Prep Kit (Illumina, CA, USA) with 150-bp paired-end on the platform (Illumina). The depth of the sequencing was larger than 6-Gb. The quality of the sequenced reads was estimated by the Q-value, then trimmed and mapped to the reference genome GRCm38 using Hisat2 (https://github.com/DaehwanKimLab/hisat2). The abundance of the annotated genes was estimated using Stringtie (version 1.3.0, Johns Hopkins University, USA), normalized with the trimmed mean of M values, and the Fragments Per Kilobase Million value was obtained on the perl script. The differentially expressed genes (DEGs) were identified with a threshold as q value < 0.05, and log2FoldChange ≥ 1.0 or ≤ -1.0. Gene ontology and KEGG enrichment was performed using the R package ‘clusterprofiler65.’ Enrichment with q value < 0.05 was considered significant. All DEGs were involved in the Gene Set Enrichment Analysis (GSEA) to investigate potential biological processes in priori defined gene sets. (http://software.broadinstitute.org/gsea/index.jsp).

### Statistical method

The results were expressed as mean ± standard deviation (SD) or percentage (%) as needed. All the data were analyzed using Prism software version 8.0 (GraphPad software, San Diego, CA, USA) or R (version 3.6.3). The QQ-plots and Shapiro–Wilk test were applied for verifying the assumptions of normality before Student’s t-test, while Mann–Whitney *U* test was used when data was abnormal distribution. A value of *P < 0.05, **P < 0.01, ***P < 0.001 were considered to be statistically significant.

## Results

### Male offspring exposed to LPS developed obesity in middle age

Prenatal LPS exposure increased the body weight in the male offspring at weaning (*P* < 0.0001) (Fig. 1B). The weight of the LPS group remained higher slightly but not significantly until 14 weeks, while a catch-up growth was observed in the normal saline (NS) group. Interestingly, as the mice grew older, the LPS group gained more weight compared with that of the control group until being sacrificed. The food intake did not differ between the two groups through the life span, although fluctuations were observed at different weeks (Fig. 1C). The body composition measurement showed increased body fat rate and decreased lean weight in LPS-treated group at 18 weeks old (NS: (8.76 ± 0.00) × 100%; LPS: (10.88 ± 0.00) × 100%, *P* < 0.05) (Fig. 1E, F), yet not at 9 weeks (Fig. 1D). After sacrifice, the anatomical parameters were recorded. Body weight (LPS: 27.956 ± 0.788; NS: 26.468 ± 0.670; *P* = 0.0065), gonadal fat (LPS: 0.475 ± 0.128; NS: 0.364 ± 0.104; *P* = 0.0486) and perirenal and retroperitoneal fat (LPS: 0.140 ± 0.043; NS: 0.080 ± 0.046; *P* = 0.0153), especially liver (LPS: 0.965 ± 0.065, NS: 0.803 ± 0.075, *P* = 0.0231), were higher in the LPS group as compared to those in the control group (Table 1).

**Table 1:** The anatomical character of the male offspring (20 weeks)

Outcomes	LPS (N = 12)^a^	NS (N = 10)	*P* value
*Anatomical character*			
Weight (g)^&^	27.956 ± 0.788	26.468 ± 0.670	0.0065^*^^*^
Quadriceps femoris (g)^&^	0.380 ± 0.031	0.426 ± 0.031	0.1923
Liver (g)^#^	0.965 ± 0.065	0.803 ± 0.075	0.0051^**^
Pancreas (g)^&^	0.241 ± 0.088	0.248 ± 0.089	0.9852
Heart (g)^&^	0.134 ± 0.012	0.145 ± 0.021	0.1554
Gonadal fat (g)^&^	0.475 ± 0.128	0.364 ± 0.104	0.0486^*^
Perirenal and retroperitoneal fat (g)^&^	0.140 ± 0.043	0.080 ± 0.046	0.0153^*^
*Biochemical character*^*b*^			
TG (mmol/gprot)	381.40 ± 20.45	256.97 ± 31.08	< .001^***^
TC (mmol/gprot)	16.94 ± 0.86	13.65 ± 1.46	< .001^***^

^a^Pregnant mice in LPS (N = 6) and NS (N = 5)

^b^N = 6 in both LPS and NS groups when conducted biochemical measurements

^&^Student’s *t* test; ^#^Mann–Whitney *U* test; *t test two-tailed *P* value (*< 0.05; **< 0.01,***< 0.001)

### Abnormal fat accumulation in the liver and adipose tissues

**Fig. 2:**
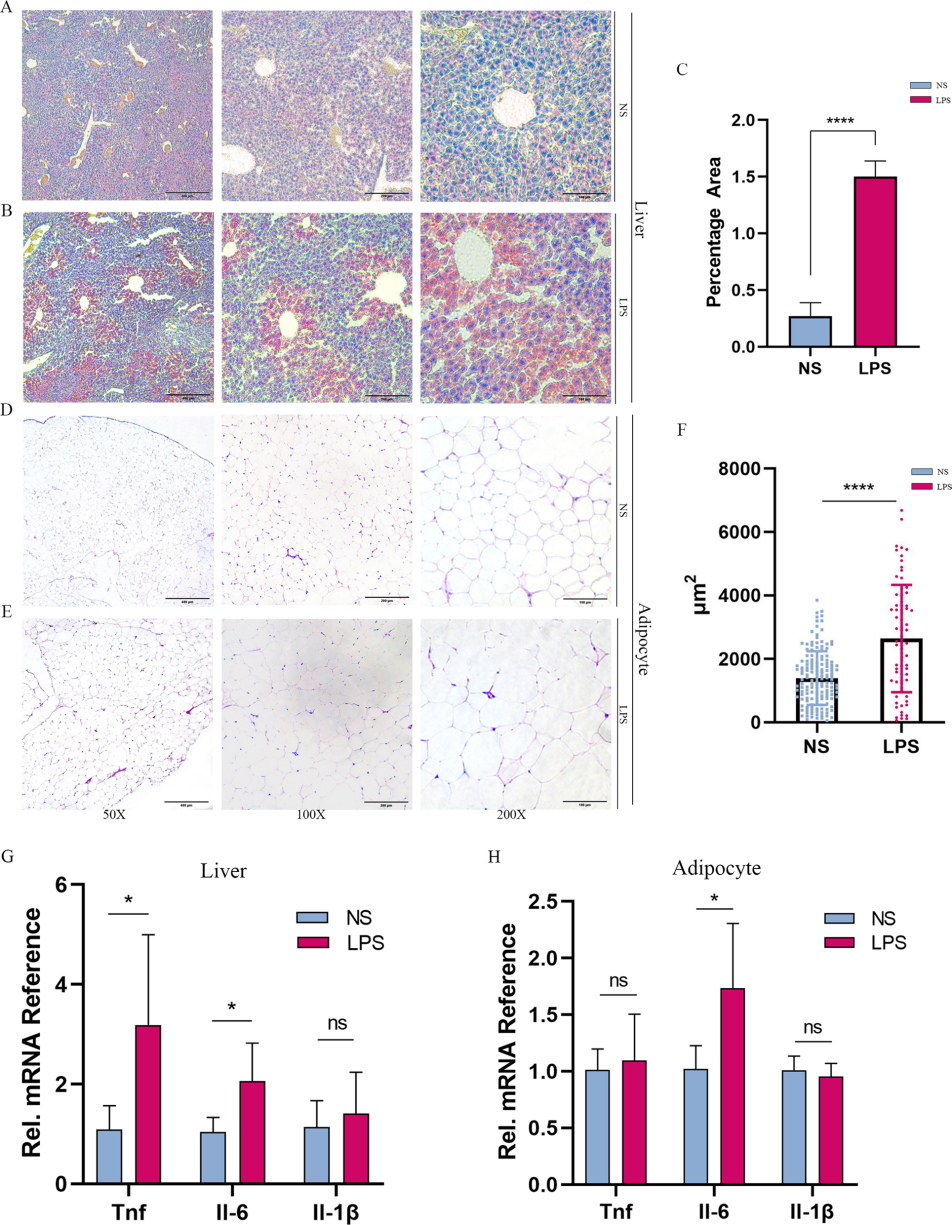
Maternal LPS exposure resulted in hepatic steatosis in male offspring. Representative H&E-treated and Oil Red O-stained sections in live of samples under 50X,100X, 200X respectively.(NS: **A**, LPS: **B**, Oil red, liver; NS: **D**, NS: **E**, H&E, adipocyte tissue). **C** Areas of oil red stained liver. **F** Areas of adipocyte. Data are mean ± SEM. *P < 0.05, **P < 0.01, ***P < 0.001 by unpaired Student’ *t* test, while oil red area with Mann–Whitney U test

As the anatomical evidence indicated, we investigated lipid metabolism in male offspring. Consistent with the obesity of the LPS group, the hepatic fatty infiltration and formation of fatty nodes were observed (Additional file 1: Figure S2 A-C). Moreover, the TC (NS: 13.65 ± 1.46; LPS: 16.94 ± 0.86 (mmol/gprot), *P* < 0.001) and TG (NS: 256.97 ± 31.08; LPS: 381.40 ± 20.45 (mmol/gprot), *P* < 0.001) levels in the liver increased (Table 1). Steatosis was confirmed by Oil-Red-O staining (NS: (0.27 ± 0.12) × 100%; LPS: (1.50 ± 0.14) × 100%, *P* < 0.0001) as well (Fig. 2A–C), without hepatocellular ballooning and abnormal liver architecture (Additional file 1: Figure S2 G-L). For adipose tissue, the size of adipocytes and the distribution of the cellular area was enlarged (NS: 1398 ± 845.2, LPS: 2642 ± 1690 (µm^2^) (Fig. 2D–F). Obesity is often associated with proinflammatory states. Next, we explored the inflammation cytokines expression in liver and fat tissues. QPCR results showed that interleukin 6 was increased in both liver and fat tissues, and TNF-α was increased in liver (Fig. 2G, H). In addition, serum TNF-α was augmented in LPS group (LPS: 63.49 ± 13.81; NS: 19.36 ± 14.78, *P* = 0.019) as determined by ELISA. However, glucose metabolism in male offspring was not affected at 9 and 18 weeks old (Additional file 1: Figure S3 A-D). Neither random nor fasting blood glucose was significantly different in two groups (Additional file 1: Figure S3 F). Glycosylated hemoglobin A1c, an indicator of the average plasma glucose concentration over a prolonged period of time, was not altered (Additional file 1,1: Figure S3 E). The results suggested the occurrence of abnormal lipid metabolism in the LPS group.

### Lipid metabolism disorders in the serum of LPS-exposed male offspring

**Fig. 3:**
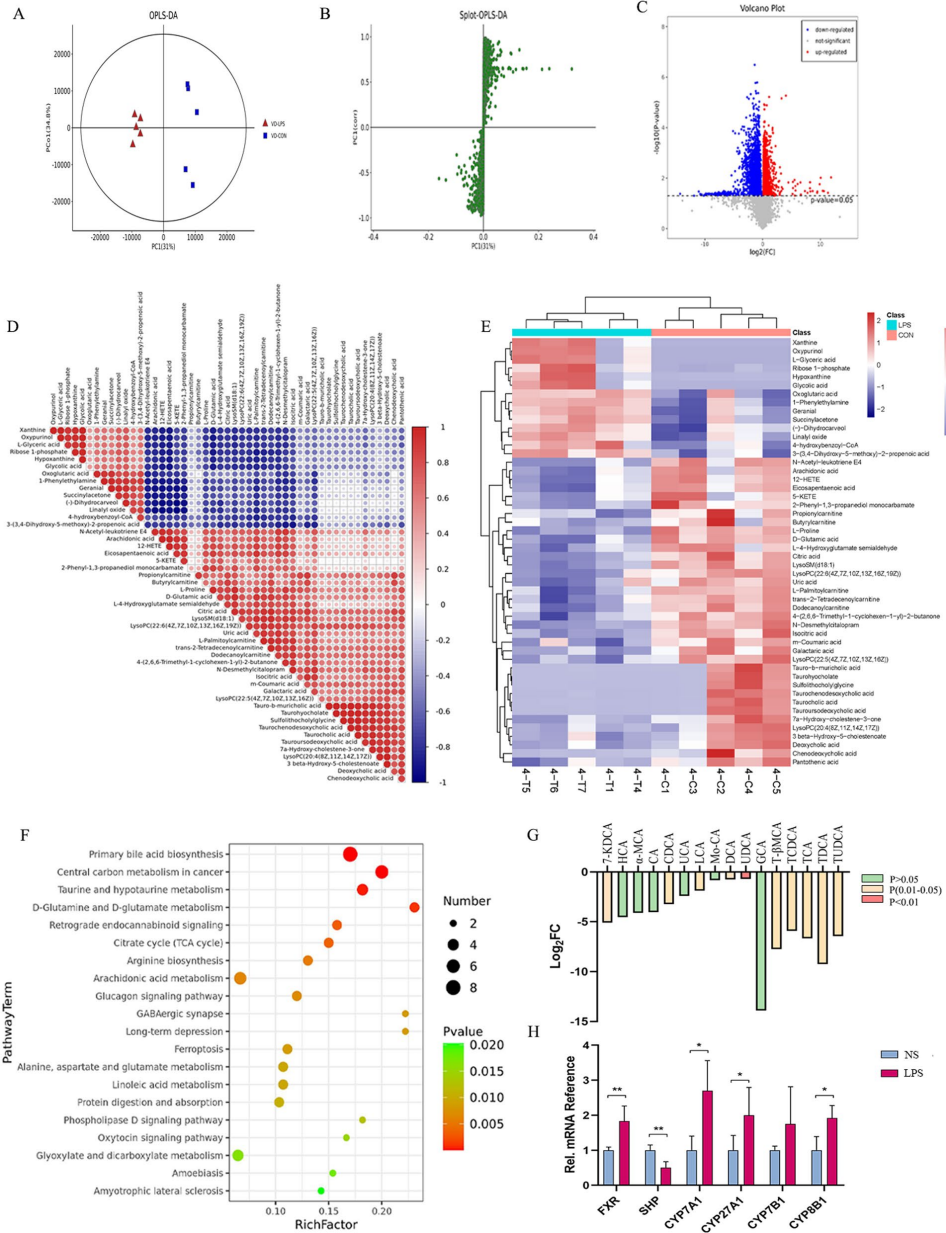
**A** Orthogonal partial least squares discriminant analysis (OPLS-DA) of metabolites (LPS: n = 5, NS: n = 5). **B** Spot of OPLS-DA. **C** Volcano map of differential metabolites. **D** Correlation analysis of differential metabolites. **E** Heat map of differential metabolites; Red indicates up-regulated genes and blue indicates down-regulated ones. **F** KEGG enrichment of differential metabolites. **G** Contents of bile acids in serum. **H** Expression of metabolic enzymes in primary bile acid synthesis. *P < 0.05; **P < 0.01; ***P < 0.0001. *FXR* Farnesoid X-activated receptor, *SHP* short heterodimer partner, *CYP7A1* cytochrome P450 family 7 subfamily A member, *CYP27A1* cytochrome P450 family 27 subfamily A member 1, *CYP8B1* cytochrome P450 family 8 subfamily B member, *Srebp1a* sterol regulatory element binding protein

**Fig. 4:**
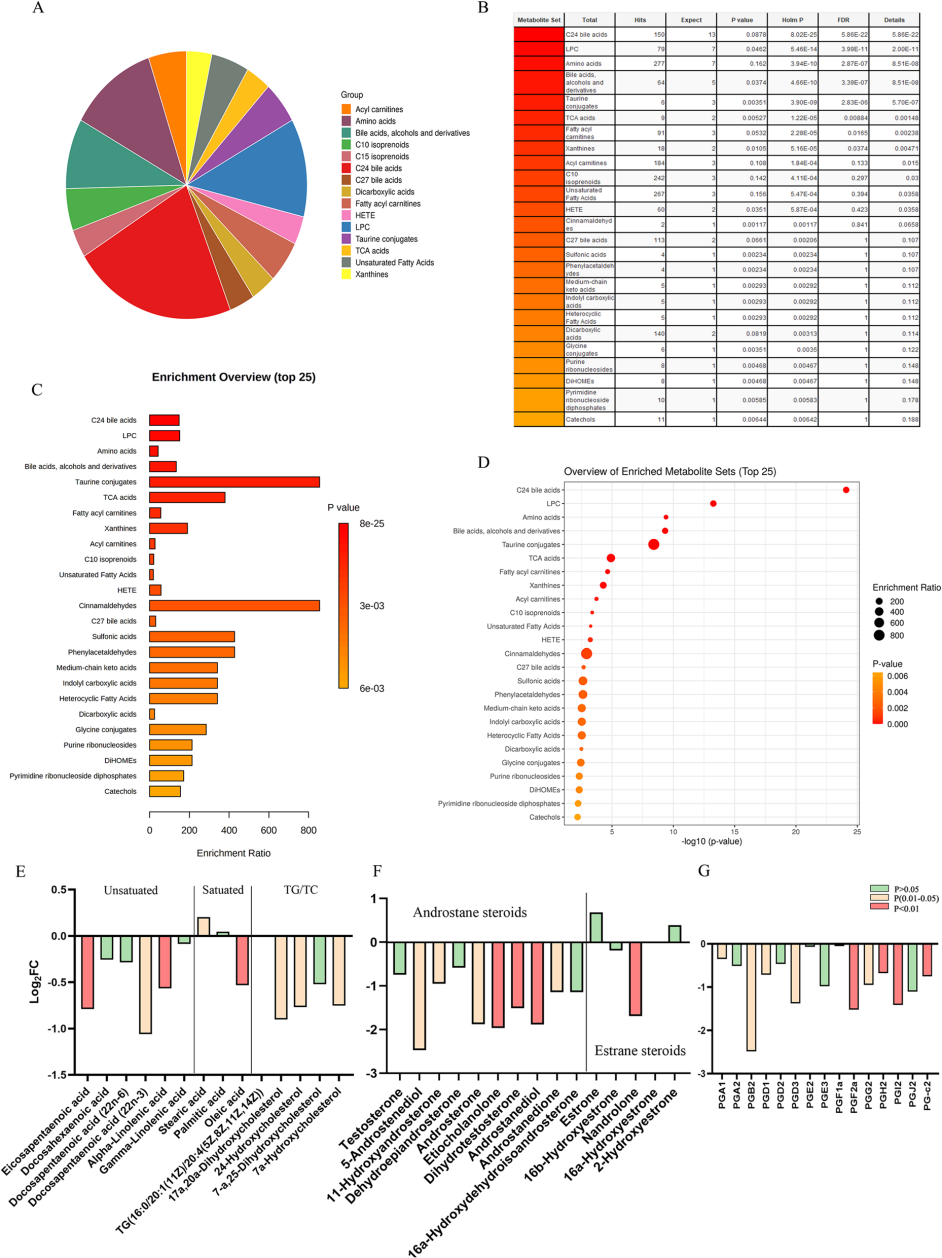
**A** Summary of the lipid constitution in differentiated metabolites. **B–D** Metabolite Set Enrichment Analysis (MSEA) overview of affected pathways. **E** The relative concentration changes of saturated and unsaturated fatty acids, **F** sex hormone, **G** Prostaglandins in serum

To further explore the metabolic disorders, a gas chromatography-mass spectrometer (GS-MS), based on untargeted metabolomics approach, was applied to the serum of mice. The primary bile acid synthesis (ID annotation: mmu00120) was enriched in Gene Ontology and KEGG analyses dramatically (Rich factor = 0.17, FDR = 4.24E−05) (Fig. 3F). The other metabolites enriched in the bile acid (BA) pathway are listed in Additional file 1: Table S4. Then, we investigated the pathway in detail and found that the serum levels of both unconjugated bile acid (UCBAs) and conjugated bile acids (CBAs) were decreased (Fig. 3G). A series of tauro-conjugated BAs consisting the majority of BAs in mice were dramatically declined, such as taurocholic acid (Fold Change (FC) = 0.0099, *P* = 0.04), taurochenodesoxycholic acid (FC = 0.0165, *P* = 0.04), taurodeoxycholic acid (FC = 0.0016, *P* = 0.04). To explore the potential reason for BAs’ decrease, we measured gene expression of several key enzymes anticipated in primary bile acids synthesis in liver, including cytochrome P450 family 7 subfamily A member 1 (CYP7A1), cytochrome P450 family 27 subfamily A member 1 (CYP27A1) in the main stream and cytochrome P450 family 8 subfamily B member 1 (CYP8B1) in the bypass. However, all these enzymes were up-regulated in LPS group, which suggested negative feedback where BA’s decrease was prior to the change in liver. Interestingly, the level of farnesoid X-activated receptor (FXR or Nr1h4), a nuclear transcription factor activated by BAs, was increased, too (Fig. 3H). Furthermore, citrate cycle (TCA cycle), arachidonic acid metabolism, and linoleic acid metabolism were enriched (Fig. 3F); the latter two unsaturated fatty acids play vital roles in many biochemical processes such as the balance of inflammation and anti-inflammation. To determine the change of the lipid composition and distribution, we isolated lipids to create a subset. The detected differentiated lipid classes (VIP > 1, *P* < 0.1) were shown in Fig. 4A. We found 59 lipid species, consisting of 13 C24 BAs, seven amino acids, seven BAs, alcohols and their derivatives and other lipid classes. Functional enrichment analyses in KEGG pathways showed the levels of C24 BAs, lysophosphatidylcholine and amino acids were significantly enriched (Fig. 4B–D). Interestingly, most unsaturated fatty acids were decreased, especially eicosapentaenoic acid (EPA) (FC = 0.579), docosapentaenoic acid (DPA) (FC = 0.480) and α-linolentic acid (FC = 0.675), which are ω-3 essential fatty acids (Fig. 4E). Moreover, all androstane steroids were decreased in the LPS group, such as etiocholanolone (FC = 0.257), dihydrotestosterone (FC = 0.351) and androstanediol (FC = 0.271) (Fig. 4F). Prostaglandins (PGs), which are lipid signals derived from arachidonic acid that are produced by cyclooxygenase enzymes and are targeted by non-steroidal anti-inflammatory drugs, were all decreased (Fig. 4G). All evidence indicated the occurrence of a lipid metabolism disorder, from triacylglycerol to cholesterol.

### Change in the transcriptome in LPS-exposed male offspring

**Fig. 5:**
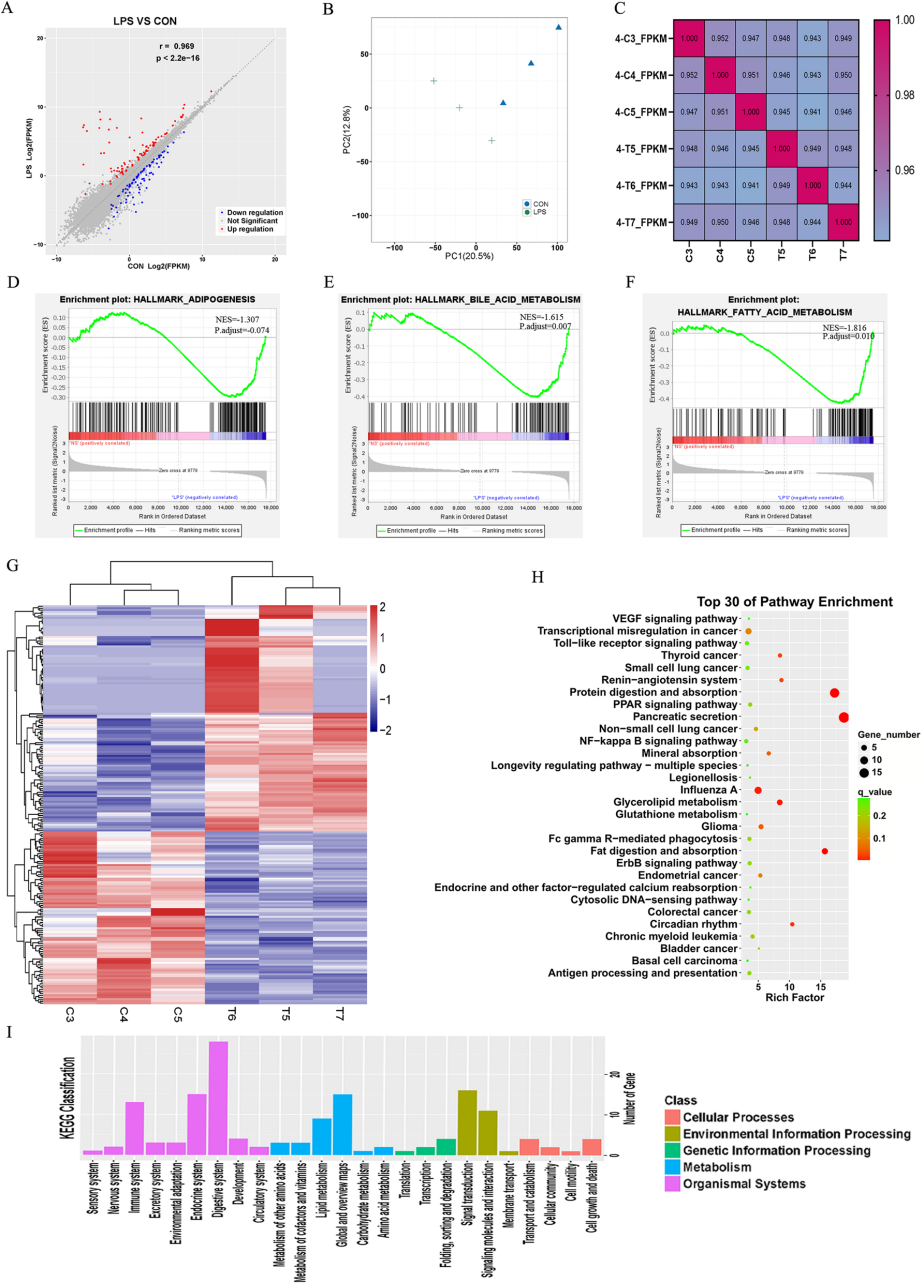
Changes in the liver transcriptional landscape of LPS exposed group compared to NS one. **A** Scattered plot of genes expression in liver; Red indicates up-regulated genes and blue indicates down-regulated ones (LPS = 3, NS = 3). **B** Principal-component analysis (PCA) of RNA-seq profiling of liver. **C** Correlation analysis of samples. **D–F** GSEA analysis of all genes. **G** Heat map of DEGs. **H, I**. GO and KEGG analysis of DEGs

**Fig. 6:**
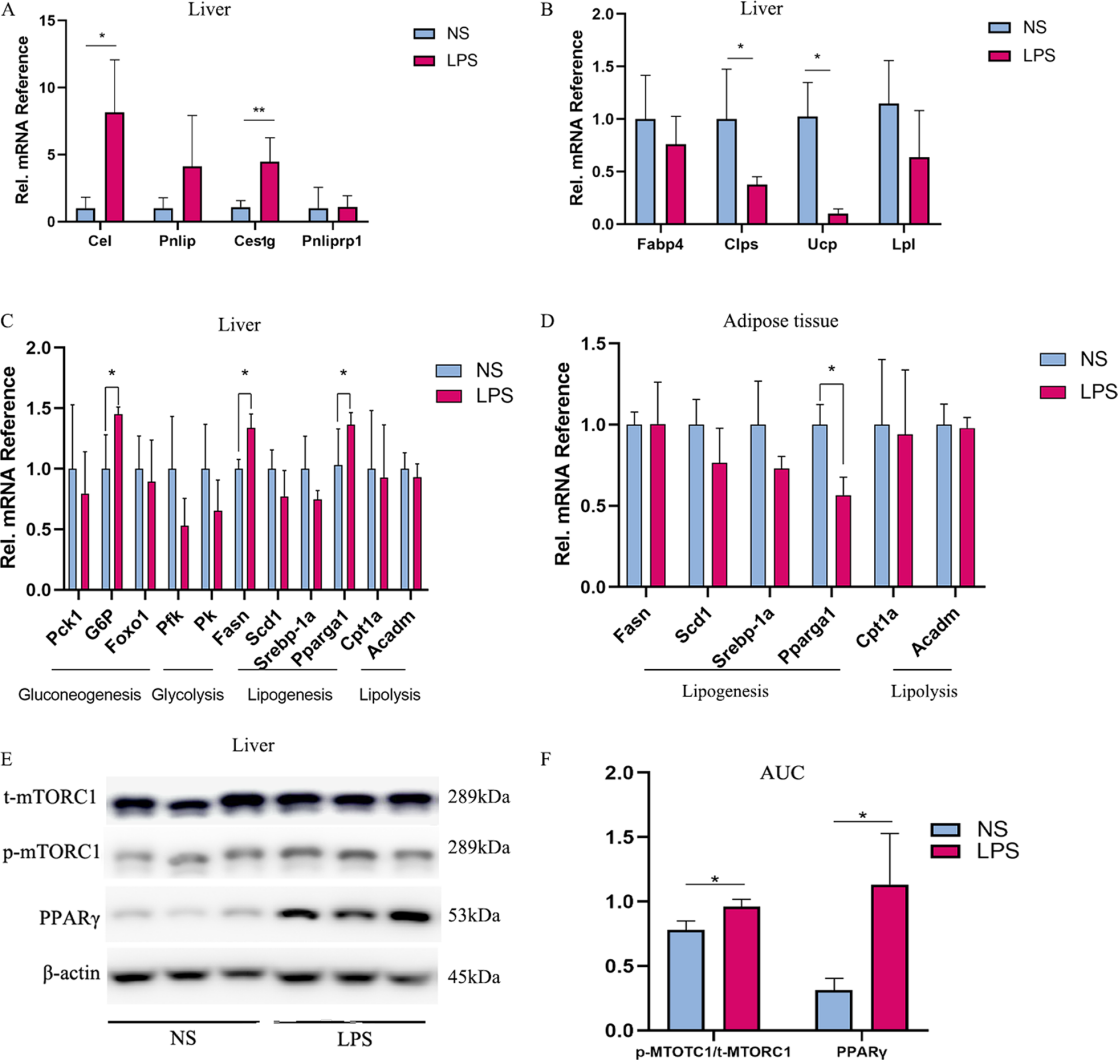
**A, B** The mRNA expression level of DEGs in RNA-seq analysis. **C, D** The mRNA expression level of glucogenesis, lipogenesis, lipid synthesis and lipolysis in liver and fat tissue mice (n = 5 per genotype). **E, F** Western blot and quantification of p-mTOR/t-mTOR, PPAR-γ, FAS, G-6-P and PCK in liver. Data are mean ± SEM. *P < 0.05, **P < 0.01, ***P < 0.001 by unpaired Student *t* test or Mann–Whitney U test. *Pla2g1b* phospholipase A2g4a, *Fabp4* fatty acid binding protein 4, *Lpl* lipoprotein lipase, *Cel* carboxyl ester lipase, *Ceslg* carboxylesterase 1G, *Fasn* fatty acid synthase, *G-6-p* glucose 6 phosphatase, *Ucp1* uncoupling protein-1, *Pnliprp1* pancreatic lipase related protein 1, *Pnlip* pancreatic lipase, *Clps* colipase

Abnormal lipid accumulation in the liver, a core organ of nutrient metabolism, was observed in previous results. Therefore, we performed RNA sequencing of the liver. When all the genes were involved, GSEA indicated the changes in adipogenesis, fatty acid metabolism and bile acid metabolism (Fig. 5D–F, Additional file 1: Table S5). KEGG analysis with a threshold of adjusted *P* value < 0.05 and fold change > 2 showed enrichment in the digestive system as pancreatic secretion was highlighted. Moreover, fat digestion and absorption were also increased in the LPS group (Fig. 5H, I). In the DEGs identified by RNA-seq, the expression of uncoupling protein-1 (*Ucp1*), a gene encoding a thermogenesis factor, was decreased (FC = 0.10 ± 0.04, *P* < 0.05); while those of carboxyl ester lipase (*Cel*) (FC = 8.15 ± 3.91, *P* < 0.05) and carboxylesterase 1G (*Ceslg*) (FC = 4.46 ± 1.79, *P* < 0.05), which are genes encoding lipid and protein digestive enzymes, were increased (Fig. 6A, B). We also explored the key enzymes of glycolipid metabolism. The increase in fatty acid synthase (*Fasn*) and glucose 6 phosphatase (*G-6-p*) levels was confirmed by RT-PCR (Fig. 6C, D). As the pathway analysis indicated, PPAR-γ, a member of the PPAR signing pathway and an upstream regulative factor of lipid metabolism (lipogenesis and cholesterol synthesis), which functioned as core metabolic regulative factor, was enriched slightly (Fig. 5H). We also examined the levels of mTOR, up-steam regulator of PPAR-γ, and found that the phosphorylation level was increased in the LPS group (Fig. 6E, F).

### Male offspring exposed to LPS consumed less energy in the rest state

**Fig. 7:**
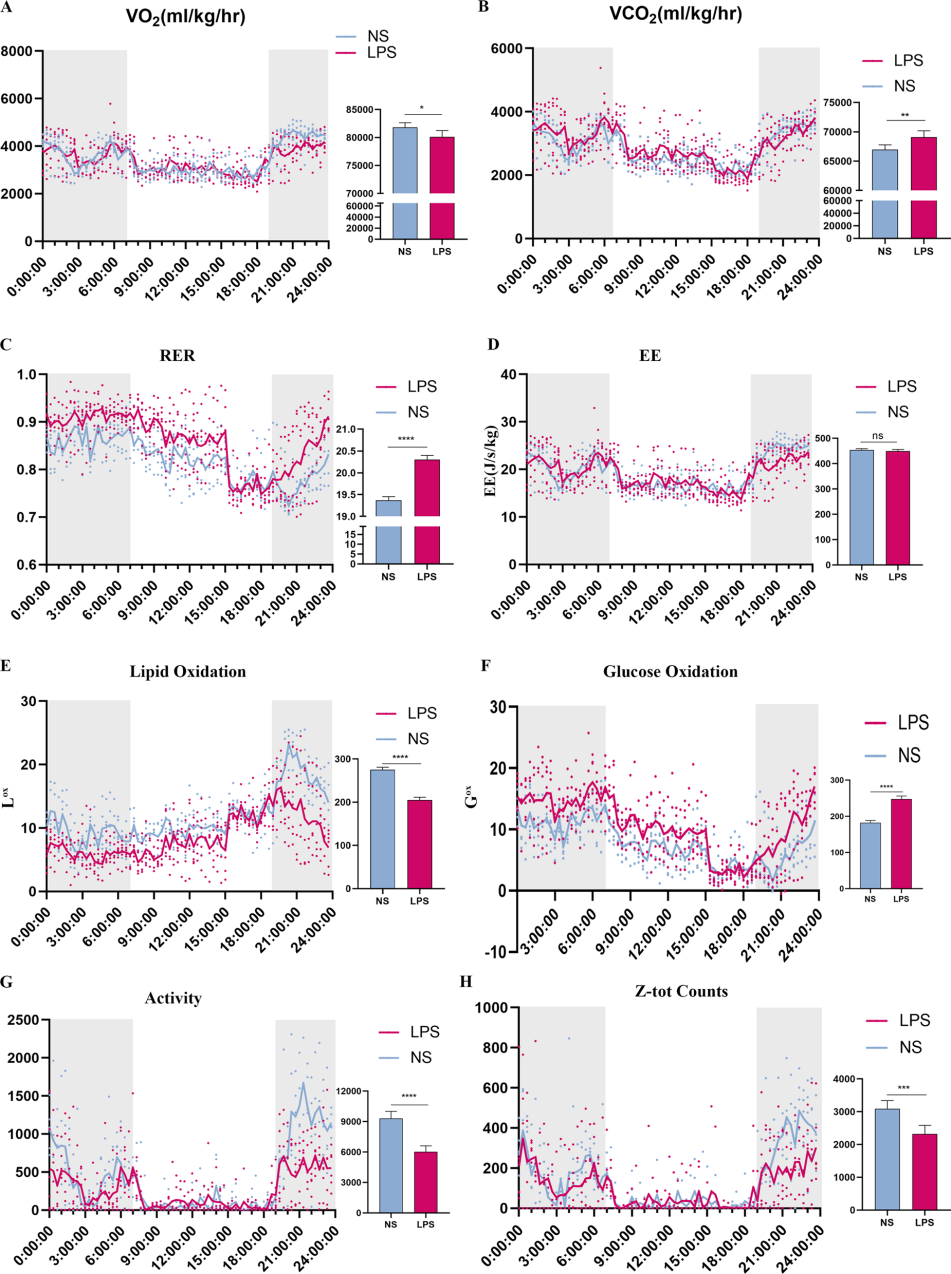
Indirect calorimetry on offspring during 24 h (LPS = 6, NS = 5) at 10 weeks. Parameters measured during 24 h for males were VO_2_ and VCO_2_ (**A**, **B**), RER (RQ, **C**), Energy expenditure (EE, **D**), Lipid oxidation (L^OX^, **E**) and carbohydrate oxidation (G^OX^, **F**). Spontaneous activity (**G**, **H**), Values were presented as mean ± SEM. *P < 0.05; **P < 0.01; ***P < 0.001; ****P < 0.0001. *RER* respiratory exchange rate, *EE* energy expenditure

**Fig. 8:**
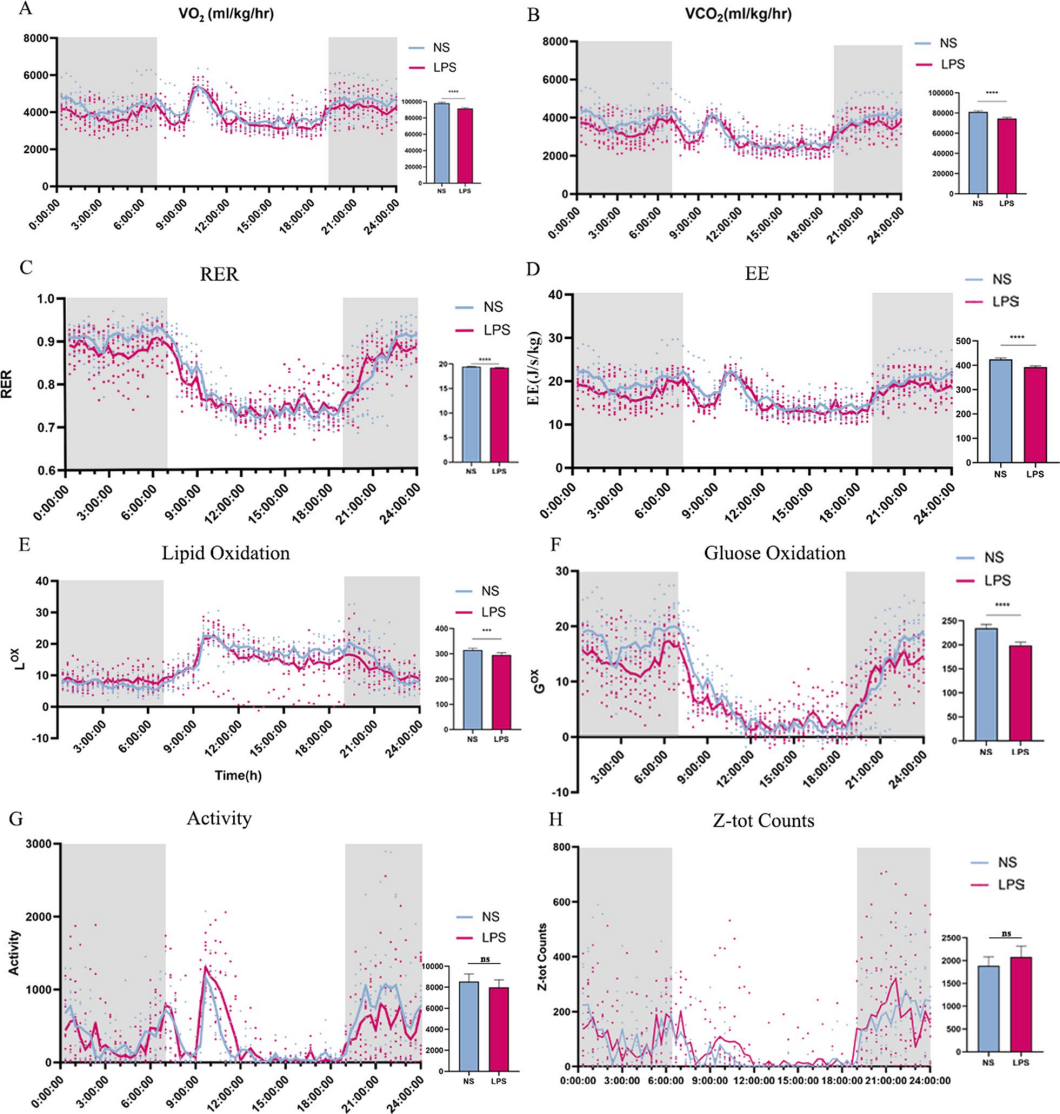
Indirect calorimetry on offspring during 24 h (LPS = 8, NS = 8) at 18 weeks. Parameters measured during 24 h for males were VO_2_ and VCO_2_ (**A, B**), RER (**C**), Energy expenditure (EE, **D**), Lipid oxidation (L^OX^, **E**) and carbohydrate oxidation (G^OX^, **F**). Spontaneous activity (**G**, **H**), Values are presented as mean ± SEM. *P < 0.05; **P < 0.01; ***P < 0.001; **** *P* < 0.0001

To determine the potential reason that contributed to the obesity, we conducted indirect calorimetry to evaluate the metabolic condition. At 9 weeks, LPS group consumed more oxygen and released less carbon dioxide, which leaded to a much higher RER (AUC: NS: 19.37 ± 0.08; LPS: 20.30 ± 0.09, *P* < 0.0001) (Fig. 7A–C). However, energy expenditure was not changed compared with that of the NS group (Fig. 7D). For further analysis, lipid oxidation (L^OX^) (AUC: NS: 275.3 ± 6.65; LPS: 204.9 ± 5.65, *P* < 0.001) was deceased, while glucose oxidation (G^OX^) (AUC: NS: 182.4 ± 6.34; LPS: 247.9 ± 8.25, *P* < 0.0001) was increased in the LPS-exposed males (Fig. 7E, F). Furthermore, more spontaneous activity was observed in LPS group (Fig. 7G, H). As grown old, the preference of energy supply seemed to change. In LPS group, oxygen consumption, carbon dioxide consumption, RER (AUC: NS: 19.4 ± 0.08; LPS: 19.22 ± 0.07), and energy expenditure (AUC:NS: 424.6 ± 5.82; LPS: 392.7 ± 5.08, *P* < 0.0001) were all decreased (Fig. 8A–D). For further analysis, both lipid oxidation (L^OX^) (AUC: NS: 314.7 ± 7.425; LPS: 295.7 ± 8.707, *P* < 0.001) and glucose oxidation (G^OX^) (AUC: NS: 234.5 ± 7.95; LPS: 198.8 ± 6.80, *P* < 0.0001) were decreased in the LPS-exposed males as compared to those of the control (Fig. 8E, F). No difference was observed in the spontaneous activity (Fig. 8G, H). The results indicated that masked endogenous factors were associated with obesity according to the energy surplus equation.

## Discussion

**Fig. 9:**
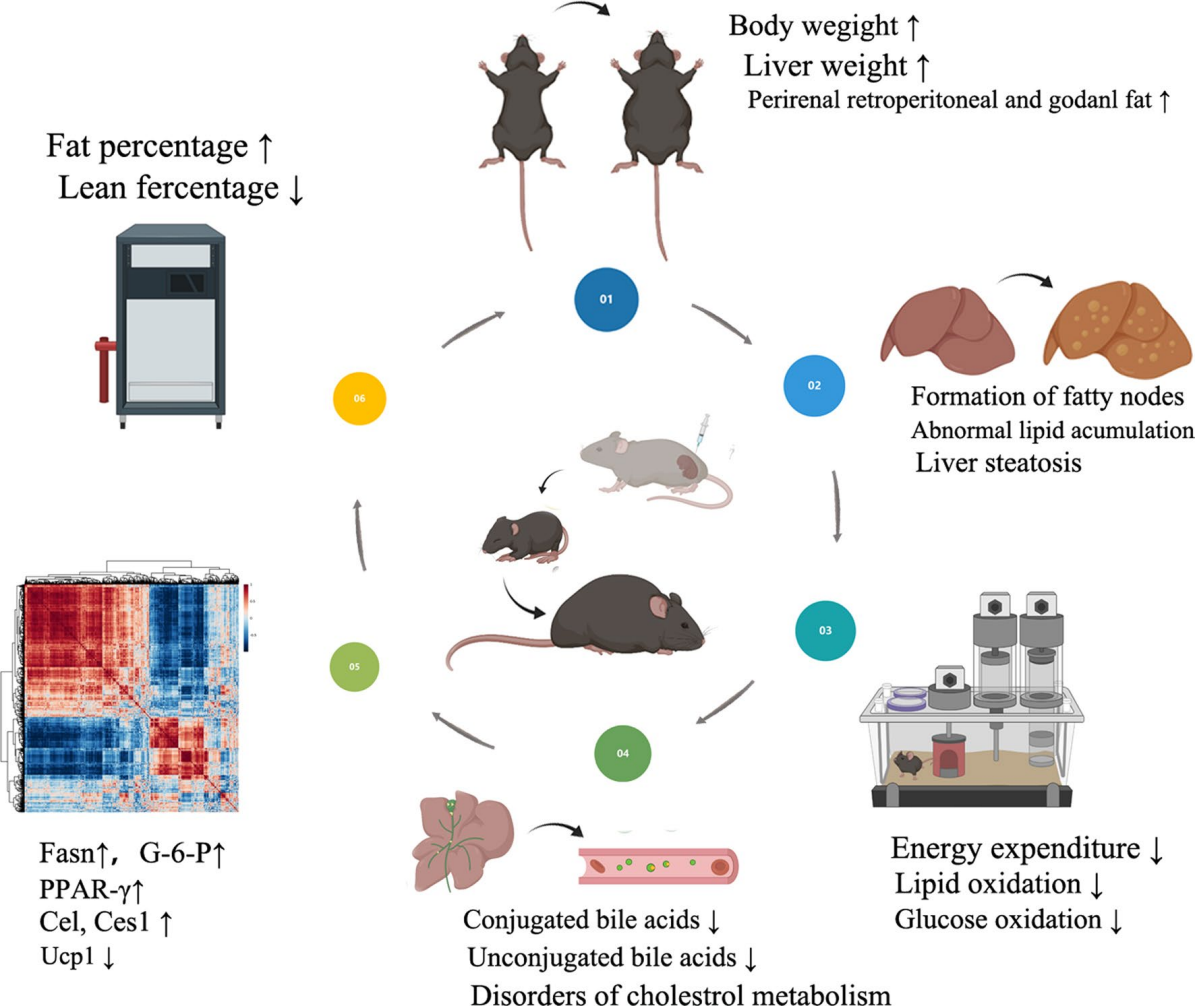
Schematic diagram of the study

In the present study, we confirmed that prenatal LPS exposure resulted in obesity, increased body fat percentage and impaired energy expenditure in middle-aged male offspring. Furthermore, abnormal lipid accumulation in the liver and adipocyte tissues was observed, accompanied with metabolites changed in serum such as unsaturated fatty acids, sex hormones and PGs. In addition, fat digestion and absorption were enriched in the LPS group in KEGG analysis. Moreover, the *Ucp1* level was decreased, while the level of *Cel *and *Ceslg* was increased in the liver. We further investigated the key enzymes of glucolipid metabolism and found that *Fasn* and *G-6-P *were upregulated. Furthermore, we found mTOR/PPAR-γ pathway, an upstream regulatory pathway of both lipid metabolism and primary BA synthesis was upregulated in the liver. The schematic diagram of the study was shown (Fig. 9).

### Obesity and energy expenditure

Prenatal LPS exposure could lead to metabolic disorders. LPS is an endotoxin produced by Gram-negative bacteria. When challenged by LPS, the type 1 immune response occurs, including secretion of inflammatory cytokines, infiltration of immune cells and subsequent adaptive response. Prenatal LPS exposure could result in abortion and prenatal morality [22, 23], and litter size might affect adult metabolism in rodents [24]. To minimize the effect of litter size, we randomly discarded pups to keep five pups in each litter. In line with our study, the exposure pregnant rats to LPS during mid-gestation led to increased body weight and adiposity in the offspring at 11 weeks old [19, 25]. In Qin’s study, the results showed the glucose level of offspring was impaired [26], while in Nilsson’s work the glucose level remained intact [25]. In general, these studies indicate that prenatal LPS exposure could result in lipid and/or glucose metabolism disorders and sometimes obesity. The discrepancies among groups may be due to differences in the dosage, time and frequency of LPS exposure.

Impaired energy expenditure might participate in the development of obesity. We decomposed the factors that might contribute to obesity as energy balance equation that energy input (EI) > energy output (EO). Then, the surplus energy is stored (ES) and converted to tissue mass (termed Ec). For two externally-modifiable components, the EI was represented by food intake. The EO, involving basal metabolic rate (BMR) and the energy spend on physical activity, was represented as an activity factor (PAF) multiplied by (BMR), as EO = PAF × BMR. Therefore, the equation for obesity becomes EI − (PAF × BMR + Ec) = dES/dt [27]. At 9 weeks old, LPS group manifested an increased respiratory exchange rate which indicated a preference for glucose as substrate for energy supply. At the same time, LPS group showed decreased spontaneous activity at night. However, the body weight, body fat percentage and energy expenditure were not affected. As grown old, no difference was found in the food intake and activity between the two groups at 18 weeks old instead of energy expenditure alternation in LPS-exposed male offspring. It was also accompanied with the decreased lipid oxidation and glucose oxidation, especially at night when rodent is physiological arousal. Interestingly, it also suggested that obesity in middle age might originate from metabolic disorders in early life. [28, 29].

### Metabolites change

Obesity in middle age of LPS exposed male offspring was companied with several metabolite change in serum such as bile acid, unsaturated fatty acid, sex hormone and prostanoid.

Bile acids are amphipathic steroid molecules and synthesized in the hepatocytes from cholesterol [30]. The rate-limiting enzymes in the bile acids synthesis includes CYP7A1, which produces the majority BA pool. Bile acid synthesis is negatively regulated via the FXR by induction of small heterodimer protein in the liver. In our study, all kinds of BAs in the serum were dramatically decreased in LPS-exposed male offspring when fasted while the expression of CYP7A1, CYP8A1 and CYP8B1 was upregulated in liver. The results indicated a secondary change from negative feedback. Bile acid has a close relationship with obesity. Researches have shown that low serum BAs are associated with fasting-induced hepatic steatosis in mice [31]. Some chemicals like theabrownin could elevate conjugated BA levels in the serum and ileum to alleviate the high-fat diet (HFD)-induced obesity [32]. However, in human, the total BA concentrations were found to be increased in obese, type II diabetic and non-alcoholic steatohepatitis patients [33]. Altogether, the discrepancy in the BA spectrum might due to the different models applied and the stage of the disease.

Omega-3 polyunsaturated fatty acids (PUFAs) have been known to be important in reducing the incidence of infection and inflammation [34–36]. Recent researches have confirmed the value of omega-3 PUFAs in reducing cardiovascular diseases and associated inflammation [37]. Interestingly, most unsaturated fatty acids like EPA, DPA, docosahexaenoic acid (DHA) and α-linolentic acid were decreased, while stearic acid, a saturated fatty acid was significantly increased in the LPS group, which indicated an inflammatory state accompanied with obesity.

PGs are derived from arachidonic acid and modulate diverse biological processes such as vascular permeability, hyperalgesia [38] and fever [39, 40]. And it is rapidly degraded and inactivated by the lung and liver after releasing and functioning locally within 1–2 mins. It is generally believed that the PG is a tissue hormone rather than a circulating hormone. Besides, PGs are affected by body type [41] and eating state [42]. In our study, decrease of all PGs might result from the reduction of arachidonic acid levels which was the precursor of all PGs, since mice were starved for 24 h before euthanasia and all unsaturated fatty acids as EPA, DHA were decreased.

Previous researches showed sex hormones may play a role as sex-dimorphic etiology in the process of obesity [43]. In our pilot experiments, obesity was not observed in female offspring exposed to prenatal LPS exposure. In addition, all androstane steroid levels were decreased in the LPS group, such as etiocholanolone, dihydrotestosterone and androstanediol, while estrogen levels were slightly increased in the LPS group. Although we did not confirm the role of sex hormone in our study due to the limitation of sample size, several researches have filled the gap. Sex hormones are related to the obesity through energy metabolism [44], body fat distribution [45] and regulation of proteins in adipose tissues [46]. A report indicated that the total testosterone and sex hormone-binding globulin were negatively associated with diabetes in male, while the result from estrogen was inverse [47]. In Xu’s work, prenatal LPS exposure led to an upregulated aromatase expression, reduced the androstanediol/estradiol ratio and altered sex hormone receptor activity in glucose metabolism disorders in middle-aged male offspring [16]. Nonetheless, how sex hormones participate in the development of obesity needs further study.

### Gene expression change

Gene Ontology and KEGG analysis of the DEGs in liver showed that pancreatic secretion was significantly enriched. The enzyme CEL, also known as bile salt-dependent or -stimulated lipase, expresses mainly in pancreatic acinar cells and performs functions of hydrolyzing fat, cholesteryl esters and fat-soluble vitamins in the duodenum [48]. When inactivated, CEL can significantly reduce susceptibility to high fat/high cholesterol diet-induced obesity [49]. *Ces1g* encodes a kind of carboxylesterase responsible for the hydrolysis of many xenobiotics and endogenous substrates involved in fatty acyl and cholesterol ester metabolism. The global loss of *Ces1/Ces1g* avoids the development of atherosclerosis by inhibiting intestinal cholesterol and triglyceride absorption [50]. However, other researches showed contradicting results, wherein *Ces1/Es-x* knockout mice resulted in increased hepatic lipogenesis and hyperlipidemia, accompanied by fat deposition in peripheral tissues [51]. Furthermore, *Ucp1* expression was decreased in the LPS group. UCP1 is a mitochondrial protein responsible for thermogenic respiration specialized in brown adipose tissue that participates in thermogenesis, temperature regulation and the regulation of energy balance. Altogether, the digestive enzymes were increased in the liver and might affect the absorption rate. At the same time, decreased expression of *Ucp1* might partly explain the impaired energy expenditure.

PPAR-γ is an important nuclear receptor that participates in lipid and cholesterol metabolism [52]. mTORC1, a core target that coordinates cell growth with the availability of nutrients, energy and growth factors participates in the PPAR-γ pathway [53, 54]. However, the function of mTOR/PPAR-γ depends on the characteristic of model. In mice fed with HFD, rosiglitazone could reduce hepatic lipid accumulation via inducing PPAR-γ [55]. Furthermore, irbesartan can ameliorate hyperlipidemia and liver steatosis by upregulating the expression of PPAR-γ, activating the AMP-activated protein kinase/protein kinase B/mTOR signaling pathway and inducing liver autophagy [56]. On the contrary, studies found resveratrol and genistein attenuate hepatic steatosis in HFD-fed mice by repressing PPAR-γ [57, 58]. In addition, ghrelin can promote lipogenesis via mTOR/PPAR-γ signaling pathway in hepatic cells [59]. In our study, PPAR-γ and p-mTOR/mTOR ratio was upregulated in LPS-exposed offspring, which might lead to the abnormal lipogenesis and accumulation in liver. Since obesity can cause and exacerbate male-factor infertility by several means [60], the change of mTOR pathway in our study might affect male reproductive system [61], which needs further research.

### Limitation and perspective

There are some limitations of present study. First, we couldn’t fully determine the role of mTOR/PPAR-γ pathway in the development of obesity, as an initiator or a responsor. Thus, future research on mice pre-treated with mTOR/PPAR-γ inhibitor is necessary. Second, endocrine profiles should be verified in the future to determine which one contributes to the obesity dominantly. Third, several metabolites have changed in our study, but few has been studied in the context of obesity. It would be interesting to explore the relationship between metabolites and obesity.

In conclusion, our study demonstrated that prenatal LPS exposure led to obesity and abnormal lipid metabolism through impaired energy expenditure.

## Supplementary Information

**Additional file 1**. **Figure S1**. Survival curves of offspring in LPS group (N = 5) compared with NS group (N = 5). **Figure S2**. **A**–**C**. Gross morphology of liver in LPS group. **D**–**F**. in NS group. **G**–**I**, Representative H&E-treated images of liver in LPS, **J**–**L**. H&E stained images of liver in NS group (40X,100X,200X, respectively). **Figure S3**. Glucose homeostasis of 9 weeks and 18 weeks old offspring. **A** Measured of GTT of males at 9 weeks old. **B** Measured of ITT f of males at 9 weeks old. **C** Measured of GTT of males at 18 weeks old. **D** Measured of ITT f of males at 18 weeks old. **E** Serum GHb1c at 20 weeks old. **F** Random and fasting blood glucose at 18 weeks. *P < 0.05, **P < 0.01, ***P < 0.0001 by unpaired Student’s *t* test. **Table S1**. Dose effects of LPS on the abortion and survival of offspring. **Table S2** .Baseline characters of mothers and offspring. **Table S3**. Sequence of primers used in RT-PCR. **Table S4**. The contents of metabolites enriched in subclass of ‘bile acids, alcohols and derivatives‘. **Table S5**. The information of gene sets enriched in GESA analysis.

## Data Availability

The data used during the study are available from the corresponding author on reasonable request. RNA-sequence data that support the findings of this study have been deposited in the GenBank database with the accession code PRJNA744538. (https://dataview.ncbi.nlm.nih.gov/object/PRJNA744538?reviewer=ik88pebm0bumssi22iavu4559a).

## Abbreviations

LPS: Lipopolysaccharide
PPAR-γ: Peroxisome proliferator-activated receptor-γ
mTORC1: Multi-component mechanistic target of rapamycin complex 1
DOHaD: Developmental origins of health and disease
GD: Gestational day
IPGTTs: Intraperitoneal glucose tolerance tests
ITTs: Insulin tolerance tests
RER: Respiratory exchange ratio
AUC: Area under the curve (AUC)
TC: Total cholesterol
TG: Triglyceride
KEGG: Kyoto Encyclopedia of Genes and Genomes
PCA: Principal component analysis
(O)PLS-DA: (Orthogonal) partial least-squares-discriminant analysis
VIP: Variable importance in the projection
DEGs: Differentially expressed genes
GSEA: Gene set enrichment analysis
FXR: Farnesoid X-activated receptor
SHP: Short heterodimer partner
CYP7A1: Cytochrome P450 family 7 subfamily A member
CYP27A1: Cytochrome P450 family 27 subfamily A member 1
CYP8B1: Cytochrome P450 family 8 subfamily B member
Srebp1a: Sterol regulatory element binding protein
AFC: Fold change
GS-MS: Gas chromatography-mass spectrometer
EPA: Eicosapentaenoic acid
DHA: Docosahexaenoic acid
BA: Bile acid
Pla2g1b: Phospholipase; A2g4a
Fabp4: Fatty acid binding protein 4
Lpl: Lipoprotein lipase
Cel: Carboxyl ester lipase
Ceslg: Carboxylesterase 1G
Fasn: Fatty acid synthase
G-6-p: Glucose 6 phosphatase
Ucp1: Uncoupling protein-1
Pnliprp1: Pancreatic lipase related protein 1
Pnlip: Pancreatic lipase
Clps: Colipase
TNF-α: Tumor necrosis factor alpha
Il-1β: Interleukin 1 beta
Il-6: Interleukin 6
EI: Energy input
EO: Energy output
ES: Energy stored
EC: Energy converted
PAF: Physical activity factor
BMR: Basal metabolic rate
HFD: High fat diet
PUFAs: Polyunsaturated fatty acids
