# Supplementary material for: Prenatal inflammation causes obesity and abnormal lipid metabolism via impaired energy expenditure in male offspring
Source: Nutr Metab (Lond). 2022 Feb 8;19:8. doi: 10.1186/s12986-022-00642-y (PMC8822840; doi:10.1186/s12986-022-00642-y)
Supplement: Supplementary file 1 — Additional file 1. Figure S1. Survival curves of offspring in LPS group (N = 5) compared with NS group (N = 5). Figure S2. A–C. Gross morphology of liver in LPS group. D–F. in NS group. G–I, Representative H&E-treated images of liver in LPS, J–L. H&E stained images of liver in NS group (40X,100X,200X, respectively). Figure S3. Glucose homeostasis of 9 weeks and 18 weeks old offspring. A Measured of GTT of males at 9 weeks old. B Measured of ITT f of males at 9 weeks old. C Measured of GTT of males at 18 weeks old. D Measured of ITT f of males at 18 weeks old. E Serum GHb1c at 20 weeks old. F Random and fasting blood glucose at 18 weeks. *P < 0.05, **P < 0.01, ***P < 0.0001 by unpaired Student’s t test. Table S1. Dose effects of LPS on the abortion and survival of offspring. Table S2 .Baseline characters of mothers and offspring. Table S3. Sequence of primers used in RT-PCR. Table S4. The contents of metabolites enriched in subclass of ‘bile acids, alcohols and derivatives‘. Table S5. The information of gene sets enriched in GESA analysis. [file 12986_2022_642_MOESM1_ESM.docx]

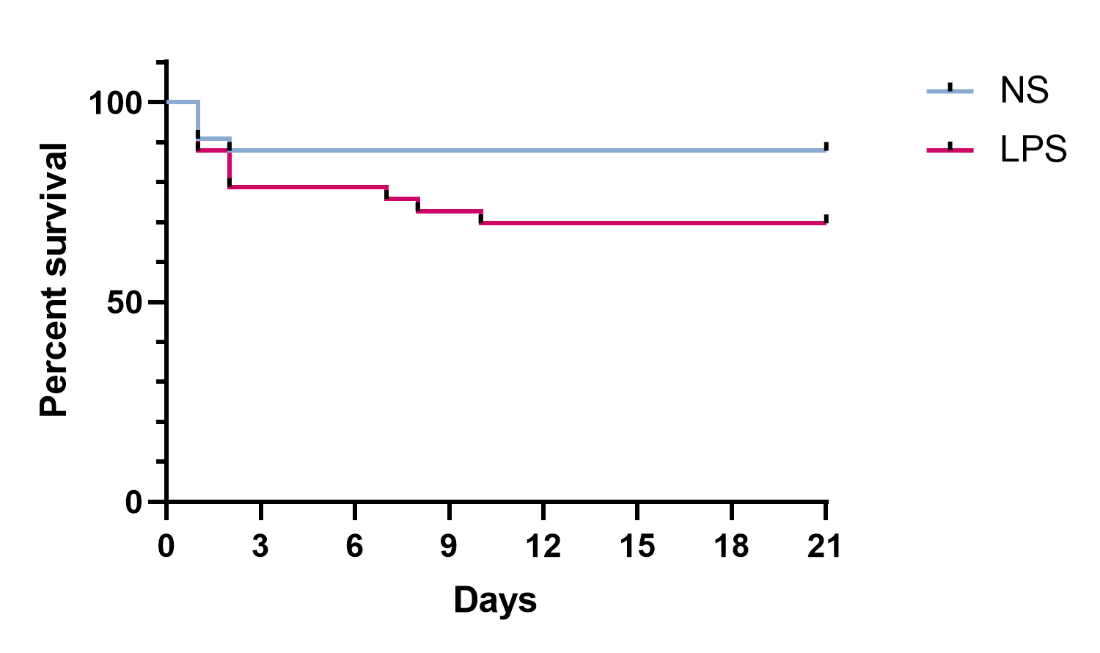


**Figure S1**. Survival curves of offspring in LPS group (N=5) compared with NS group (N=5).


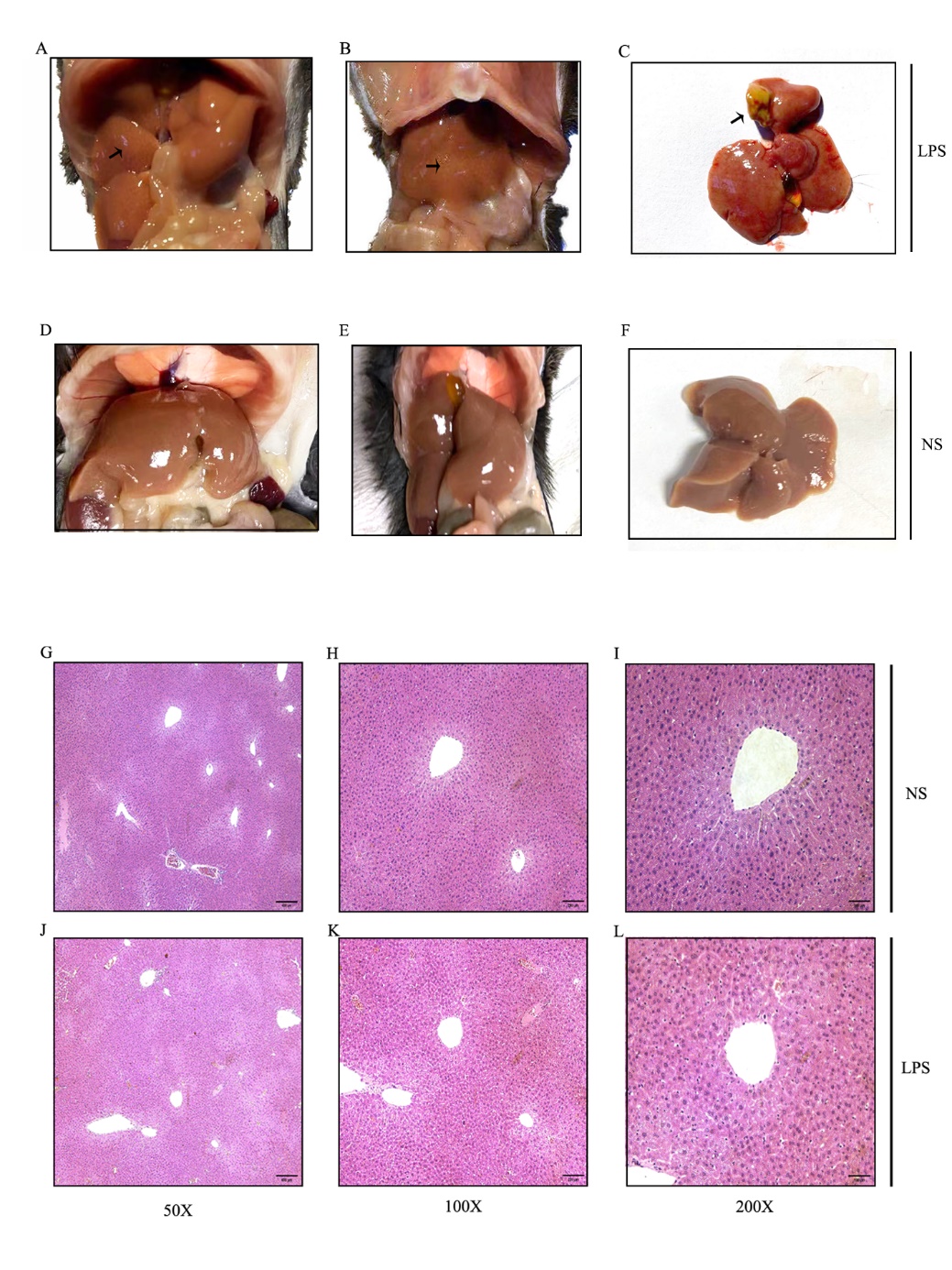


**Figure S2. A,B,C**. Gross morphology of liver in LPS group. **D,E,F**. in NS group. **G,H,I,** Representative H&E-treated images of liver in LPS, **J,K,L**. H&E stained images of liver in NS group (40X,100X,200X, respectively).


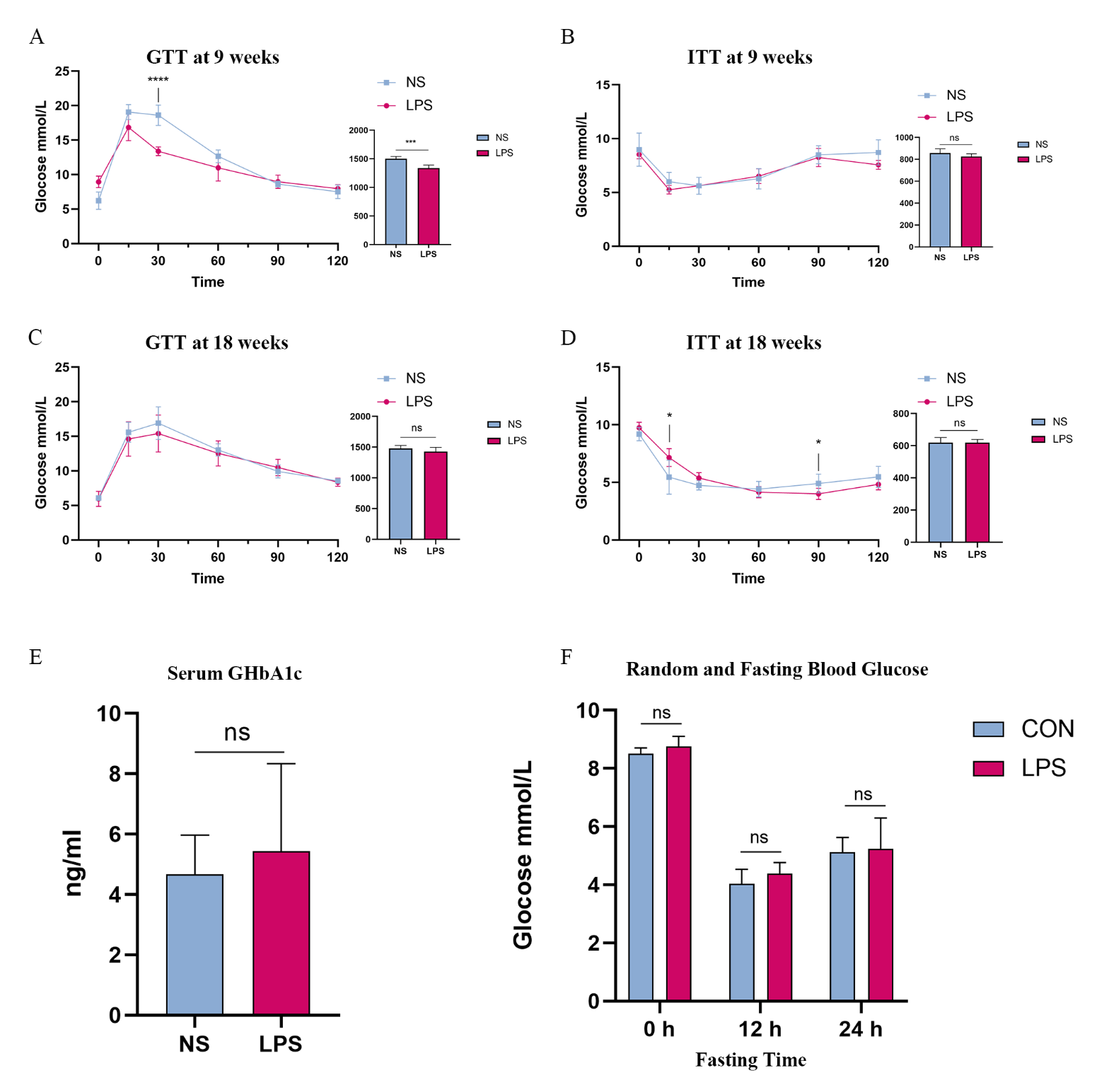


**Figure S3.** Glucose homeostasis of 9 weeks and 18 weeks old offspring. **A**. Measured of GTT of males at 9 weeks old. **B.** Measured of ITT f of males at 9 weeks old. **C**. Measured of GTT of males at 18 weeks old. **D**. Measured of ITT f of males at 18 weeks old. **E**. Serum GHb1c at 20 weeks old. **F**. Random and fasting blood glucose at 18 weeks. **P* < 0.05, ***P* < 0.01, ****P* < 0.0001 by unpaired Student’s t-test.

**Table S1. Dose effects of LPS on the abortion and survival of offspring**

| Pregnant mice | Dose of LPS injection (µg/kg) | Abortions | Births | | Survival (%) | |  |
| --- | --- | --- | --- | --- | --- | --- | --- |
| (number) | GD15^a^ | (%) | | (number) | | at 3 weeks old | |
| 2 | 500 | 100% (2/2) | | 0 | | 0% (0/0) | |
| 2 | 250 | 50% (1/2) | | 1 | | 0% (0/1) | |
| 2 | 100 | 50% (1/2) | | 1 | | 0% (0/1) | |
| 3 | 50 | 33% (1/3) | | 10 | | 80% (8/10)^b^ | |
| 3 | 25 | 0% (0/3) | | 20 | | 90% (18/20) | |

^a^ gestational day

^b^ The abortion cases were excluded

**Table S2. The characters of mothers and offspring**

|  | LPS | NS | *P*-value |
| --- | --- | --- | --- |
| Pregnant mice number | 6 | 5 | - |
| Birth number per litter | 6.7 ± 1.5 | 8.5 ± 2.1 | 0.158 |
| Male (%) per litter | 44.1 ± 21.1 | 60.1 ± 18.3 | 0.201 |
| Gestational age (days) per litter | 19.3 ± 0.5 | 20.4 ± 0.5 | 0.008^**^ |
| Survival (%)  at weaning per litter | 77.5 ± 22.0 | 81.3 ± 19.1 | 0.779 |

**P* < 0.05, ***P* < 0.01, ****P* < 0.0001 by unpaired Student t test.

**Table S3. Sequence of primers used in RT-PCR**

| **Gene Symbol** | | **Forward** | **Reverse** |
| --- | --- | --- | --- |
| **β-actin** | - | CGTGCGTGACATCAAAGAGAAG | CGTTGCCAATAGTGATGACCTG |
| **G6P** | Glucose-6-phosphatase | CCATGGGCGCAGCAGGTG | AGGTAGATCCGGGACAGACAGACG |
| **Pck1** | Phosphoenolpyruvate carboxykinase-1 | CGCTGGATGTCGGAAGAG | AGTCTGTCAGTTCAATACCAATC |
| **Foxo1** | Forkhead box O1 | AAGAGTTAGTGAGCAGGCTACAT | TTCCCAATGGCACAGTCCTT |
| **Pfk** | Phosphofructokinase | GCCATCGCCGTGTTGAC | GCCCTGACGGCAGCATT |
| **PK** | Pyruvate kinase | CCCATCACGGCCCGCAACACT | ATTCAGCCGAGCCACATTCATTCC |
| **Acadm** | Acyl-Coenzyme A dehydrogenase, medium chain | ATGCCTGTGATTCTTGCTGGA | ACATCTTCTGGCCGTTGATAAC |
| **Scd1** | Stearoyl CoA desaturase-1 | TTCTTGCGATACACTCTGGTGC | CGGGATTGAATGTTCTTGTCGT |
| **PPARγ1** | Peroxisome proliferator activated receptor γ1 | TGAAAGGGCCAAACAGAGAG | GTAAATCACACGGCGCTCTT |
| **Srebp1a** | Sterol regulatory element binding protein 1 A | AACGTCACTTCCAGCTAGAC | CCACTAAGGTGCCTACAGAGC |
| **Cpt1a** | Carnitine palmitoyltransferase 1a (Liver) | GCGAAGTGTCGGCAGACCTA | TGTTCCGATTCGTCCAACGT |
| **Fasn** | Fatty acid synthase | CAAATACAATGGCACCCTGA | TGGCGAAGCCGTAGTTAGTT |
| **Fxr** | Farnesoid X-activated receptor | TCCAGGGTTTCAGACACTGG | GCCGAACGAAGAAACATGG |
| **Nr0b2 (Shp-1)** | nuclear receptor subfamily 0, group B, member 2 (Short heterodimer partner) | CGATCCTCTTCAACCCAGATG | AGGGCTCCAAGACTTCACACA |
| **Cyp7a1** | Cytochrome P450 family 7 subfamily A member 1 | AGCAACTAAACAACCTGCCAGT | ACTAGTCCGGATATTCAAGGATGCA |
| **Cyp27a1** | Cytochrome P450 family 27 subfamily A member 1 | AGGGCCTCACATCAACAGAG | GCTGACGCTGTAGGACACAT |
| **Cyp7b1** | Cytochrome P450 family 7 subfamily B member 1 | GGAGCCACGACCCTAGATG | TGCCAAGATAAGGAAGCCAAC |
| **Cyp8b1** | Cytochrome P450 family 8 subfamily A member 1 | GGGAGTGGGTGGAAGTGAG | GTCCTGCATGGATGAAGCT |
| **Cel** | Carboxyl ester lipase | ACAACACCTATGG GCAAGAAG | CTCCTCCCCGTCAT ACAGGTA |
| **Pnliprp1** | Pancreatic lipase related protein 1 | GCAGAACTGGGTG GTTGACAT | CGTGTAGGTAGTCTGAGAGCCT |
| **Pnlip** | Pancreatic lipase | CTGGGAGCAGTAGCTGGAAG | AGCGGGTGTTGATCTGTGC |
| **Clps** | Colipase | GAACAGTATGCAGTGTAAGAGCA | GCAGATGCCATAGTTGGTGTTG |
| **Pla2g1b** | Phospholipase A2g4a | GTGTGGCAGTTCCGCAATATG | CCTGTCTAAGTCGTCCACTGG |
| **Fabp4** | Fatty acid binding protein 4 | AAGGTGAAGAGCATCATAACCCT | TCACGCCTTTCATAACACATTCC |
| **Lpl** | Lipoprotein lipase | CAGAGTTTGACCGCCTTCC | AATTTGCTTTCGATGTCTGAGAA |
| **Ucp1** | Uncoupling protein 1 | ATACTGGCAGATGACGTCCC | GTACATGGACATCGCACAGC |
| **Tnf** | Tumor necrosis factor | CAGGCGGTGCCTATGTCTC | CGATCACCCCGAAGTTCAGTAG |
| **Il-1β** | Interleukin 1 beta | TTCAGGCAGGCAGTATCACTC | GAAGGTCCACGGGAAAGACAC |
| **Il-6** | Interleukin 6 | TCTATACCACTTCACAAGTCGGA | GAATTGCCATTGCACAACTCTTT |

**Table S4. The contents of metabolites enriched in subclass of ‘bile acids, alcohols and derivatives‘**

| **Metabolites** | **Sub Class** | **KEGG** | **Score** | ***P*-value** | **adj. *P*-value** | **log2(FC)** | **Change** |
| --- | --- | --- | --- | --- | --- | --- | --- |
| Taurine | Organosulfonic acids and derivatives | C00245 | 49 | 0.0191 | 0.0875 | 0.1917 | Up |
| Chenodeoxycholic acid | Bile acids, alcohols and derivatives | C02528 | 42.3 | 0.0491 | 0.1391 | -3.2387 | Down |
| Taurocholic acid | Bile acids, alcohols and derivatives | C05122 | 54.5 | 0.0424 | 0.1315 | -6.6520 | Down |
| 3a,7a-Dihydroxy-5b-cholestan-26-al | Bile acids, alcohols and derivatives | C05445 | 49.4 | 0.0141 | 0.0746 | -0.9167 | Down |
| 7a-Hydroxy-cholestene-3-one | Cholestane steroids | C05455 | 38.1 | 0.0094 | 0.0616 | -0.9183 | Down |
| Taurochenodesoxycholic acid | Bile acids, alcohols and derivatives | C05465 | 55.9 | 0.0404 | 0.1297 | -5.9179 | Down |
| Tetrahydrocortisol | Bile acids, alcohols and derivatives | C05465 | 42.8 | 0.0003 | 0.0230 | -0.9708 | Down |
| 5b-Cyprinol sulfate | Bile acids, alcohols and derivatives | C05468 | 38.1 | 0.0446 | 0.1329 | -9.3284 | Down |
| 24-Hydroxycholesterol | Bile acids, alcohols and derivatives | C13550 | 37.3 | 0.0319 | 0.1147 | -0.7686 | Down |
| Taurodeoxycholic acid | Bile acids, alcohols and derivatives | C05463 | 56 | 0.0427 | 0.1317 | -9.2469 | Down |
| Taurochenodesoxycholic acid | Bile acids, alcohols and derivatives | C05465 | 55.9 | 0.0404 | 0.1297 | -5.9179 | Down |
| Nutriacholic acid | Bile acids, alcohols and derivatives |  | 55.5 | 0.0984 | 0.2144 | -4.7540 | Down |
| Tauroursodeoxycholic acid | Bile acids, alcohols and derivatives |  | 54.8 | 0.0408 | 0.1300 | -6.4285 | Down |
| Taurocholic acid | Bile acids, alcohols and derivatives | C05122 | 54.5 | 0.0424 | 0.1315 | -6.6520 | Down |
| Tauro-b-muricholic acid | Bile acids, alcohols and derivatives |  | 53.9 | 0.0483 | 0.1380 | -7.7483 | Down |
| Taurohyocholate | Bile acids, alcohols and derivatives | C15516 | 52.9 | 0.0453 | 0.1334 | -6.5113 | Down |
| Cholic acid | Bile acids, alcohols and derivatives | C00695 | 52.5 | 0.0853 | 0.1960 | -4.0384 | Down |
| 7-Ketodeoxycholic acid | Bile acids, alcohols and derivatives |  | 51.7 | 0.0446 | 0.1329 | -5.0953 | Down |
| 3-Oxocholic acid | Bile acids, alcohols and derivatives |  | 51.1 | 0.0827 | 0.1922 | -4.5793 | Down |
| Sulfolithocholylglycine | Bile acids, alcohols and derivatives | C11301 | 50.9 | 0.0454 | 0.1334 | -7.5661 | Down |
| 3a,6a,7b-Trihydroxy-5b-cholanoic acid | Bile acids, alcohols and derivatives | C17727 | 50.9 | 0.0987 | 0.2146 | -4.6969 | Down |
| 3a,7a-Dihydroxy-5b-cholestan-26-al | Bile acids, alcohols and derivatives | C05445 | 49.4 | 0.0141 | 0.0746 | -0.9167 | Down |
| Deoxycholic acid | Bile acids, alcohols and derivatives | C04483 | 49.3 | 0.0133 | 0.0727 | -0.7471 | Down |
| Glycocholic acid | Bile acids, alcohols and derivatives | C01921 | 48.7 | 0.0774 | 0.1846 | -13.8877 | Down |
| 3a,6b,7b-Trihydroxy-5b-cholanoic acid | Bile acids, alcohols and derivatives | C17726 | 47.2 | 0.0573 | 0.1521 | -7.8952 | Down |
| Alpha-Muricholic acid | Bile acids, alcohols and derivatives | C17647 | 45.6 | 0.0879 | 0.2000 | -4.1176 | Down |
| Hyocholic acid | Bile acids, alcohols and derivatives |  | 44.6 | 0.0920 | 0.2055 | -4.5323 | Down |
| Tetrahydrocortisol | Bile acids, alcohols and derivatives | C05465 | 42.8 | 0.0003 | 0.0230 | -0.9708 | Down |
| Chenodeoxycholic acid | Bile acids, alcohols and derivatives | C02528 | 42.3 | 0.0491 | 0.1391 | -3.2387 | Down |
| Ursocholic acid | Bile acids, alcohols and derivatives | C17644 | 41.2 | 0.0588 | 0.1547 | -2.4094 | Down |
| Chenodeoxycholic acid glycine conjugate | Bile acids, alcohols and derivatives | C05466 | 38.9 | 0.0425 | 0.1315 | -6.3486 | Down |
| Isohyodeoxycholic acid | Bile acids, alcohols and derivatives |  | 38.8 | 0.0182 | 0.0856 | -0.6545 | Down |
| 7alpha-Hydroxy-3-oxo-4-cholestenoate | Bile acids, alcohols and derivatives | C17337 | 38.6 | 0.0041 | 0.0442 | -0.6129 | Down |
| 3 alpha,7 alpha,26-Trihydroxy-5beta-cholestane | Bile acids, alcohols and derivatives | C05444 | 38.5 | 0.0178 | 0.0844 | -0.6288 | Down |
| 3beta,7alpha-Dihydroxy-5-cholestenoate | Bile acids, alcohols and derivatives | C17335 | 38.4 | 0.4156 | 0.5663 | -0.2438 | Down |
| Isoursodeoxycholic acid | Bile acids, alcohols and derivatives | C17662 | 38.4 | 0.0465 | 0.1354 | -4.5020 | Down |
| Hyodeoxycholic acid | Bile acids, alcohols and derivatives |  | 38.1 | 0.0529 | 0.1452 | -4.7933 | Down |
| Sulfolithocholic acid | Bile acids, alcohols and derivatives |  | 38.1 | 0.1858 | 0.3304 | -0.1881 | Down |
| 5b-Cyprinol sulfate | Bile acids, alcohols and derivatives | C05468 | 38.1 | 0.0446 | 0.1329 | -9.3284 | Down |
| Lithocholyltaurine | Bile acids, alcohols and derivatives | C02592 | 38.1 | 0.0509 | 0.1417 | -26.9231 | Down |
| Murocholic acid | Bile acids, alcohols and derivatives | C15515 | 37.7 | 0.0786 | 0.1863 | -0.8184 | Down |
| Ursodeoxycholic acid | Bile acids, alcohols and derivatives | C07880 | 37.7 | 0.0068 | 0.0547 | -0.6938 | Down |
| Lithocholic acid | Bile acids, alcohols and derivatives | C03990 | 37.7 | 0.0104 | 0.0640 | -1.8894 | Down |
| Isodeoxycholic acid | Bile acids, alcohols and derivatives | C17661 | 37.7 | 0.0253 | 0.1009 | -0.9097 | Down |
| 2-Deoxycastasterone | Bile acids, alcohols and derivatives | C15793 | 37.6 | 0.0128 | 0.0714 | -0.5167 | Down |
| 24-Hydroxycholesterol | Bile acids, alcohols and derivatives | C13550 | 37.3 | 0.0319 | 0.1147 | -0.7686 | Down |
| 27-Deoxy-5b-cyprinol | Bile acids, alcohols and derivatives | C05446 | 37.2 | 0.0088 | 0.0601 | -3.2498 | Down |
| 6-Deoxocastasterone | Bile acids, alcohols and derivatives | C15802 | 37.1 | 0.0080 | 0.0577 | -1.1775 | Down |
| Biliverdin | Bilirubins | C00500 | 37.7 | 0.0012 | 0.0308 | -1.2538 | Down |
| Bilirubin | Bilirubins | C00486 | 52.5 | 0.0051 | 0.0482 | -1.2219 | Down |

**Table S5. The information of gene sets enriched in GESA analysis**

| **Gene set name** | **Enrichment Score (ES)** | **Normalized Enrichment Score (NES)** | **FDR(q-value)** | **Regulation(LPS *vs* NS)** |
| --- | --- | --- | --- | --- |
| Oxidative Phosphorylation | -0.42865667 | -1.8974255 | 0.009756098 | UP |
| Fatty Acid Metabolism | -0.43093583 | -1.8157544 | 0.007317073 | UP |
| Bile Acid Metabolism | -0.40465102 | -1.6153705 | 0.01612466 | UP |
| Peroxisome | -0.33070034 | -1.3467153 | 0.07229675 | UP |
| Adipogenesis | -0.29822245 | -1.3071227 | 0.0736748 | UP |
| Interferon Gamma Response | 0.5896975 | 1.7522099 | 8.660714E-4 | Down |
| Inflammatory Response | 0.581686 | 1.7347934 | 8.573809E-4 | Down |
| Tnfa Signaling Via Nfkb | 0.5721993 | 1.7224569 | 6.430357E-4 | Down |
| Il6 Jak Stat3 Signaling | 0.60497546 | 1.7173438 | 5.1442854E-4 | Down |
| Interferon Alpha Response | 0.56398153 | 1.6045448 | 0.0031624755 | Down |

The top 5 enrichment pathways were shown in both regulated directions.

FDR < .05 was considered significant.
